# Comparative analysis and visualization of multiple collinear genomes

**DOI:** 10.1186/1471-2105-13-S3-S13

**Published:** 2012-03-21

**Authors:** Jeremy R Wang, Fernando Pardo-Manuel de Villena, Leonard McMillan

**Affiliations:** 1 Department of Computer Science, University of North Carolina at Chapel Hill, Chapel Hill, NC, USA; 2 Department of Genetics, Lineberger Comprehensive Cancer Center, University of North Carolina at Chapel Hill, Chapel Hill, NC, USA

## Abstract

**Background:**

Genome browsers are a common tool used by biologists to visualize genomic features including genes, polymorphisms, and many others. However, existing genome browsers and visualization tools are not well-suited to perform meaningful comparative analysis among a large number of genomes. With the increasing quantity and availability of genomic data, there is an increased burden to provide useful visualization and analysis tools for comparison of multiple collinear genomes such as the large panels of model organisms which are the basis for much of the current genetic research.

**Results:**

We have developed a novel web-based tool for visualizing and analyzing multiple collinear genomes. Our tool illustrates genome-sequence similarity through a mosaic of intervals representing local phylogeny, subspecific origin, and haplotype identity. Comparative analysis is facilitated through reordering and clustering of tracks, which can vary throughout the genome. In addition, we provide local phylogenetic trees as an alternate visualization to assess local variations.

**Conclusions:**

Unlike previous genome browsers and viewers, ours allows for simultaneous and comparative analysis. Our browser provides intuitive selection and interactive navigation about features of interest. Dynamic visualizations adjust to scale and data content making analysis at variable resolutions and of multiple data sets more informative. We demonstrate our genome browser for an extensive set of genomic data sets composed of almost 200 distinct mouse laboratory strains.

## Background

Genome browsers are one of the most common bioinformatics tools used by biologists. Browsers allow biologists to visualize genomic features such as genes, SNPs, CG islands, transcription factor binding sites, and many others and to place these features in their genomic context. They are also useful in adding and viewing genome annotations and feature-specific information. Generally, genome browsers support analysis of a single genome, but there is often a need to compare features between one or more genomes. Existing tools are not well-suited to doing this. Many visualization methods have been developed to support comparative genomics of animals from different species. These include phylogenetic trees, alignment viewers, Circos diagrams [1], and dot-matrix methods [2]. Tools which perform comparative analysis include BLAST (pairwise alignment analysis) [3] and VISTA [4]. Generally these methods support only comparisons between a small number of genomes. There is a need for comparative analysis and visualization tools supporting members of the same species with largely collinear genomes. Our goal was to develop a system which supports simultaneous and dynamic analysis of many (10 s to 100 s) collinear genomes.

A web-based resource for investigating genomic data from multiple samples simultaneously would aid many common comparative genome analyses including disease association studies and expression analysis. Our system supports any generic genomic data set, allowing it to be an extensible framework for analysis, not simply a data resource. Like existing genome browsers and viewers, we represent different categories of genomic data as horizontal tracks covering a particular region of the genome. Unlike previous work, we use color to better indicate important regions and facilitate more intuitive comparison. In addition, we allow dynamic sorting and local reordering of tracks.

Comparison between genomes of different samples of the same species, particularly the analysis of local haplotype and phylogeny, can provide insight into gene origins and individual variations. They also aid in understanding population structure. Understanding local genomic variations and population structure is the key to studies of individual genes and their association with disease. We need to be able to not only determine similarities and differences between samples genome-wide, but also at the level of individual loci.

There are many genome browsers and viewers that can integrate multiple data sets pertaining to a particular genome sequence whether it is specific or a species consensus. Many of these are standalone desktop applications. There also exist several web-based genome browsers. These browsers, including the UCSC genome browser [5], GBrowse [6], Ensembl [7], NCBI Map Viewer [8], and JBrowse [9], display multiple tracks of data and support a variety of useful navigation techniques that allow the genome to be traversed and visualized at various resolutions. However, existing browsers are limited in their ability to support dynamic and comparative analysis between multiple genomes.

The UCSC Genome Browser [5] is the standard and most prevalent web-based genome browser. The UCSC browser originally targeted the human genome data as a part of the Human Genome Project. It has since been extended to numerous other species. The goal of the UCSC browser is to make a particular set of data broadly accessible and navigable. It does not focus on any particular analysis but is a comprehensive resource for integrating, displaying, and navigating publicly accessible genome data. The browser supports standard functions including navigation by panning and zooming. Data sets of interest can be displayed in tracks and reordered manually by the user. The UCSC browser functions as a window into very comprehensive sets of data for many different species, but does not support comparisons between either inter- or intraspecific genomes. The UCSC browser does not support dynamic interactions with the displayed data. Instead, pages must be reloaded in their entirety any time that new data is requested. Due to this limitation, data retrieval is necessarily limited to a small window or few data types to allow quick and easy analysis.

The Generic Genome Browser (GBrowse) [6] is another widely used web-based genome browser available for human, mouse and other model organisms. The main difference between GBrowse and the UCSC browser is extensibility. GBrowse is designed to be extended with new and user-provided data sets, and as such it provides a flexible framework for displaying and navigating arbitrary genome information. Otherwise, GBrowse uses the same basic navigation and display structure as the UCSC browser. Data sets can be individually selected and are displayed as horizontal tracks stacked on top of one another and aligned to a common genomic scale. Unlike the UCSC browser, GBrowse supports asynchronous retrieval and navigation of data, meaning the entire page does not need to be reloaded to update the genomic regions displayed. This reduces the computational overhead on both the server and client, refreshing only those parts that need to be changed. However, GBrowse is limited in its ability to display small-scale details at high resolutions. Since the representation and visualization of data is essentially fixed, fine details such as SNPs are often omitted when viewing large regions.

The Ensembl genome database project [7], a joint venture between the Sanger Institute and the European Bioinformatics Institute (EBI), was initiated with a goal of providing full genome data along with various annotation as a public resource for researchers. The Ensembl genome browser serves as a publicly available web-based browser for this data. Although initially focusing on the human genome, the browser now includes many model-organism genomes with annotations including genes, DNA and RNA alignments, and many other annotations. The browser function itself is very similar to the UCSC browser, supporting traditional navigation techniques. Ensembl also uses asynchronous data requests to retrieve data when it is needed. In addition, detailed annotations and links to more thorough information are displayed when a feature such as a gene or contig is selected.

The National Center for Bioinformatics Information (NCBI) provides the NCBI Map Viewer [8] as an online tool for browsing genomes. Unlike others, the NCBI Map Viewer displays the genome vertically with tracks for only the assembly, contigs, and genes while focusing on detailed description and annotation for these features linking to other useful NCBI tools for directly accessing related genes, SNPs, proteins, and more. Map Viewer also does not provide any dynamic navigation mechanism, therefore the entire page must be reloaded each time the genome window is adjusted. The browser serves best as a hub through which other resources are accessed by genomic position and is not a viable analysis tool by itself.

JBrowse (Javascript-based genome browser) [9] is a more recent web-based tool designed to allow navigation and analysis of genomes and is available as a framework that researchers can set up and fill with their own data. JBrowse takes advantage of the dynamic features available to modern browsers and allows for more interactive and dynamic visualizations. JBrowse's focus is on supporting dynamic and fluid transitions between displayed windows, for example showing a smooth sliding transition as a user pans in one direction along the genome. JBrowse also supports client-side dynamic rendering rather than the server-side image or block rendering as in most other browsers. This reduces the server-side computation and time and cost associated with data transfer of full images between the server and client. JBrowse leverages the computational power on the user's browser to draw and dynamically shift and rescale the visualization, leading to a more intuitive understanding of the relationship between genomic features as a user shifts the frame of reference. JBrowse is a good tool for generic genome annotation analysis, but, as with other existing browsers, it does not provide suitable techniques for visualizing multiple genomes concurrently.

Existing genome browsers are well suited for generic genome annotation and are useful for analysis of the specific data sets they are tailored to, but there are many limitations. Available data is essentially static. In many cases, users have the ability to customize the browser to use different data or display only what they are interested in, but the underlying information representation remains constant. The visualization is essentially static, where the current region of interest is shipped to the viewer. Data can be viewed at multiple resolutions, but no further attempt is made to improve upon the usability of the visualization for a particular purpose. It is hard to quickly glean information and understanding from the visualization. These tools are frequently used to provide access to publicly available data sources rather than to support novel visualizations for analysis. Our browser addresses the following limitations of existing genome browsers: it supports simultaneous exploration of multiple aligned genomes, it allows for dynamic rearrangements of tracks to support comparisons, and it provides alternative visualization modes based on the current displayed scale.

## Methods

### Design

Our genome browser is available as a public website allowing users to view, explore, and analyze multiple genomic data without requiring a standalone application http://msub.csbio.unc.edu. Data is stored on the web server and the client side consists of only the web browser. It has been tested and works on most modern web browsers and operating systems. Tested browsers include Chromium/Google Chrome 10.0, Firefox 3.6, Firefox 4.0, Internet Explorer 8, and Safari 5.0. It even loads on iPhones (iOS 4.2.1). Platform interoperability and constant availability make it an easy and useful tool for genetic analysis.

**Figure 1:**
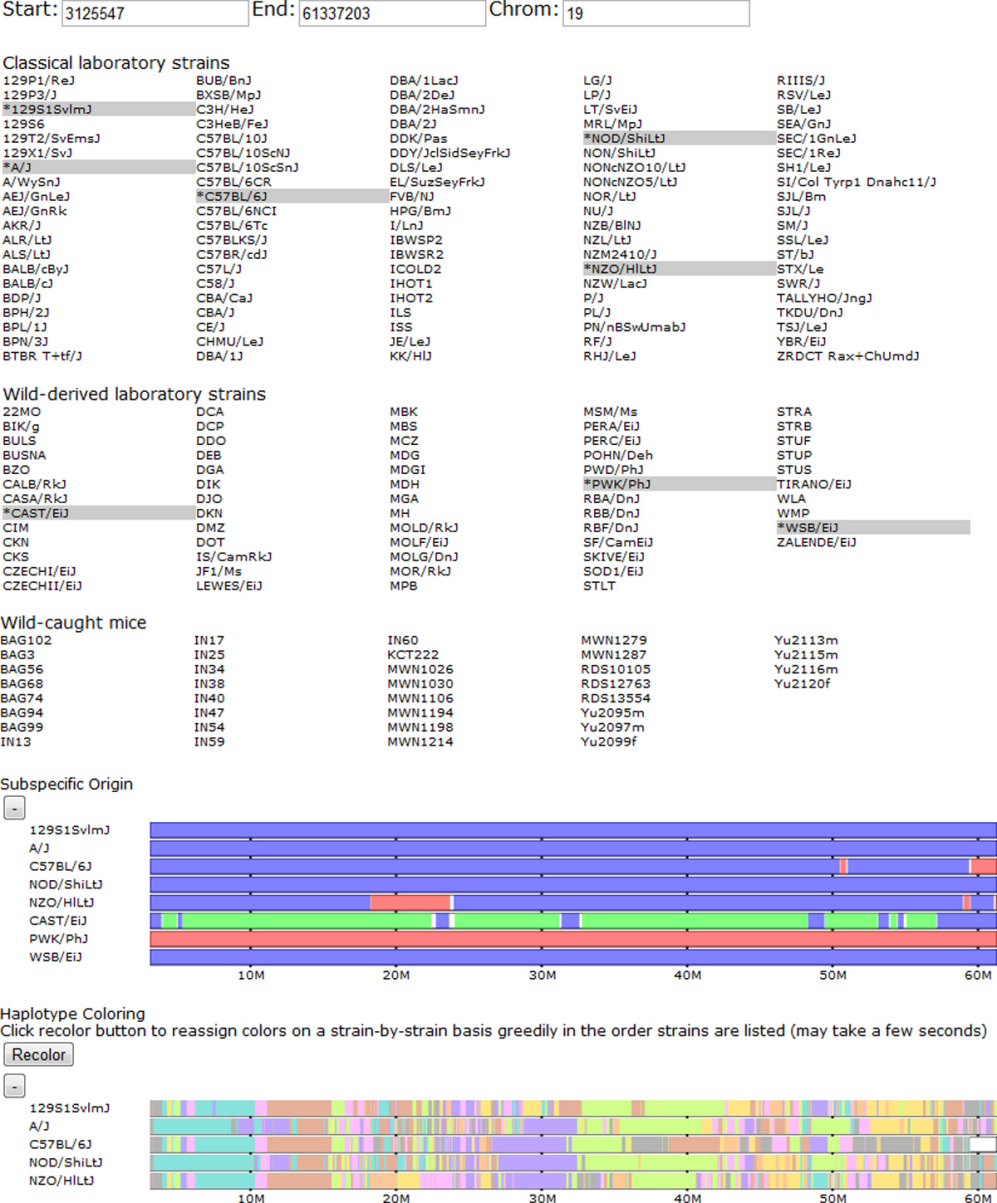
**Browser overview.** Shown from top to bottom are the browser's viewing window editor, strain selection panel, and data tracks for subspecific origin and haplotype similarity. The user selects a subset of genomes, which are highlighted and then displayed as tracks on one of several groups. Some data sets, such as the haplotype coloring, show only data for the selected classical laboratory strains. The user can drag tracks within a group to reorder the display of samples. This reordering is reflected in all grouped tracks. Users can navigate the genome by manually entering positions or use the navigation buttons to zoom in and out and pan side to side across the genome. Data tracks can also be collapsed and expanded using the +/- button above each track. In the subspecific origin group the colored tracks indicate the subspecies origin of each strain. Throughout, blue indicates *Mus musculus domesticus*, red indicates *M. m. musculus*, and green indicates *M. m. castaneus*. Shared colors in the haplotype tracks indicate a common haplotype.

**Figure 2:**
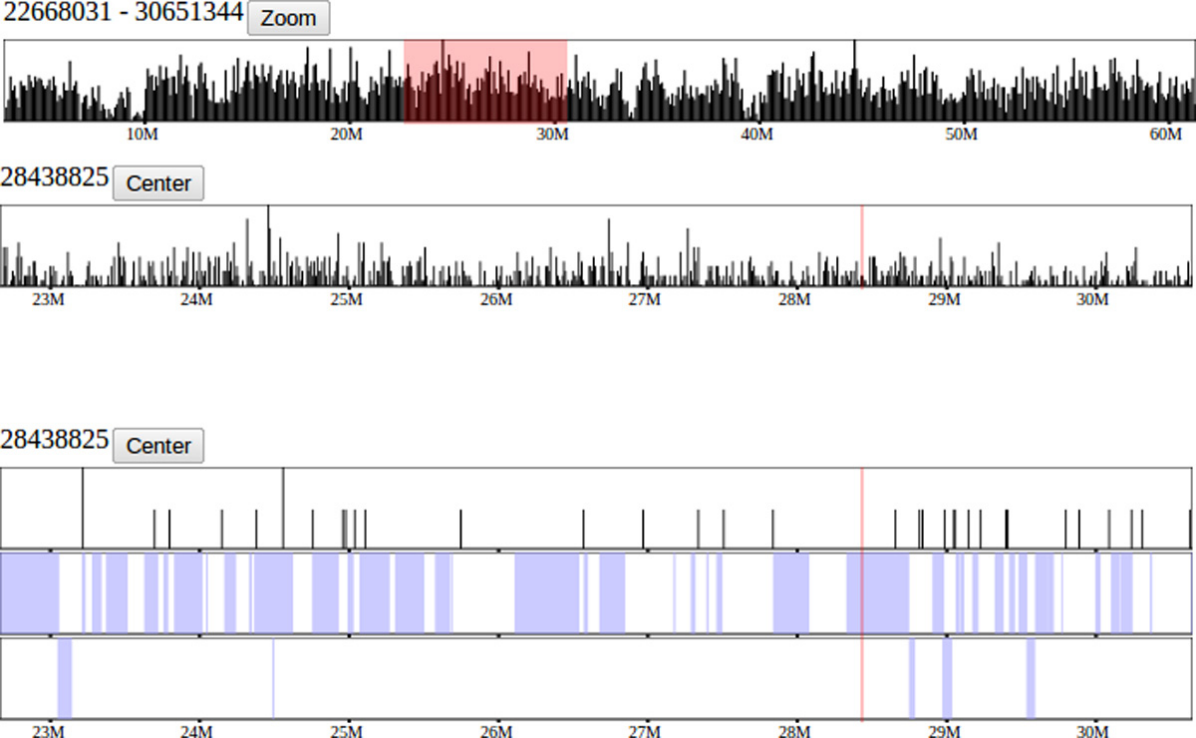
**Highlighting and zooming.** The topmost track shows a region of mouse chromosome 19 selected by clicking and dragging across the genome. The region is highlighted on all tracks and the user is given the option to zoom in to the chosen region. The results of zooming into the highlighted regions are displayed in the following 5 tracks. The bottom two tracks show a single point in the genome selected to allow the user to center the viewing window on the chosen position. The position selected by clicking one track is reflected in all other tracks to pinpoint aligned features.

Our basic visualization techniques are similar to existing browsers and genome viewers in that separate types of genomic data are represented as vertically stacked horizontal bars (tracks) covering a selected region of the genome (Figure 1). We support various navigation techniques including manually selecting a region of the genome, panning backward and forward through the genome, and zooming in and out. Clicking and dragging over any track highlights the region over which the pointer is dragged over all displayed tracks. This allows users to highlight regions of interest to easily compare between track groups. In addition, once a region is selected, a button appears to allow zooming in to the selected region such that it fills the entire viewing window (Figure 2). This allows for precise navigation to features of interest. There are also navigation buttons to zoom out by fixed small (two) and large (ten) ratios of the displayed window size. Panning side to side is supported by four buttons, two pan in each direction, one a short distance and one a long distance, 10% and 50% of the viewing window size, respectively. Panning small distances allows the user to fine tune the display to focus on a region of interest. Further panning allows users to scan the genome for nearby features while maintaining a local frame of reference. In addition to panning and zooming, when a point on any track is clicked, a vertical cursor line is highlighted to allow visual alignment of features at that point. The display window may then be recentered around the selected position to best show the chosen feature and its surrounding area (Figure 2). The displayed samples are selected or deselected by clicking the strain name in the selector region. Data sets can also be individually shown or hidden depending on the analysis performed by clicking to toggle the show/hide button next to each track group.

We provide easy access to the data underlying the visualization through the browser interface. For most data types, the displayed information can be retrieved as a delimited text file by clicking the output button below each track, which retrieves the underlying data for the currently selected sets of active genomes and within the displayed window so that no further filtering is required.

The basic data representation used by our browser is a set of possibly overlapping intervals specified by their genome coordinates (typically chromosome and position). Intervals are displayed as horizontal blocks that are displayed along the viewing window based on the bounding positions of the interval. If intervals are smaller than the display resolution, they are presented as histograms. Overlapping intervals can also be displayed on subsequent stacked tracks. This data representation supports a wide variety of genome annotations and allows the browser to be easily extended to novel data sets.

**Figure 3:**
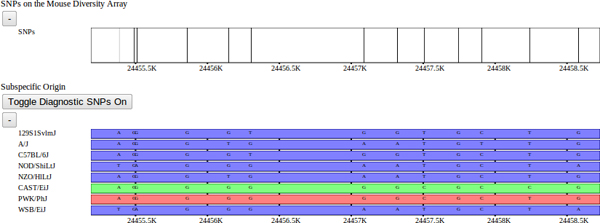
**SNPs and subspecific origin.** Mouse Diversity Array SNPs (above) and subspecific origin (below) shown at a fine resolution. A high density of SNPs is represented as a histogram across the genome. As the user zooms in, the histogram's bar heights dynamically adapt to display the relative SNP densities in each genomic region. Individual SNPs are displayed as vertical ticks along the SNP track as the display resolution approaches an individual base-pair. Alleles for each strain are shown overlaying the subspecific origin to enable detailed analysis. Alleles are also shown overlaid on the haplotype track at low resolutions (not shown).

The visualization method used changes dynamically based on both content and scale. Unlike previous browsers, the visualization changes to aid analysis based on the subset of samples chosen, the order of samples, and the viewing resolution of the data. When examining a small region of fine detail, features such as SNPs and genotype intervals are displayed as discrete blocks or points such that individual features and their exact relationship can be determined. At coarse levels of detail, when there are too many small-scale features to accurately display them individually, compound or consensus visualizations, such as a histogram, are shown, providing a more useful high level interpretation of the data (Figure 3). Dynamic visualization techniques are also applied to the subset and order of comparative samples selected. Samples can be displayed simultaneously and their tracks reordered such that they can be easily compared. In addition, a variety of visualizations are computed dynamically based on the current subset of samples selected, such as intervals of sequence identity among the selected set of samples.

**Figure 4:**
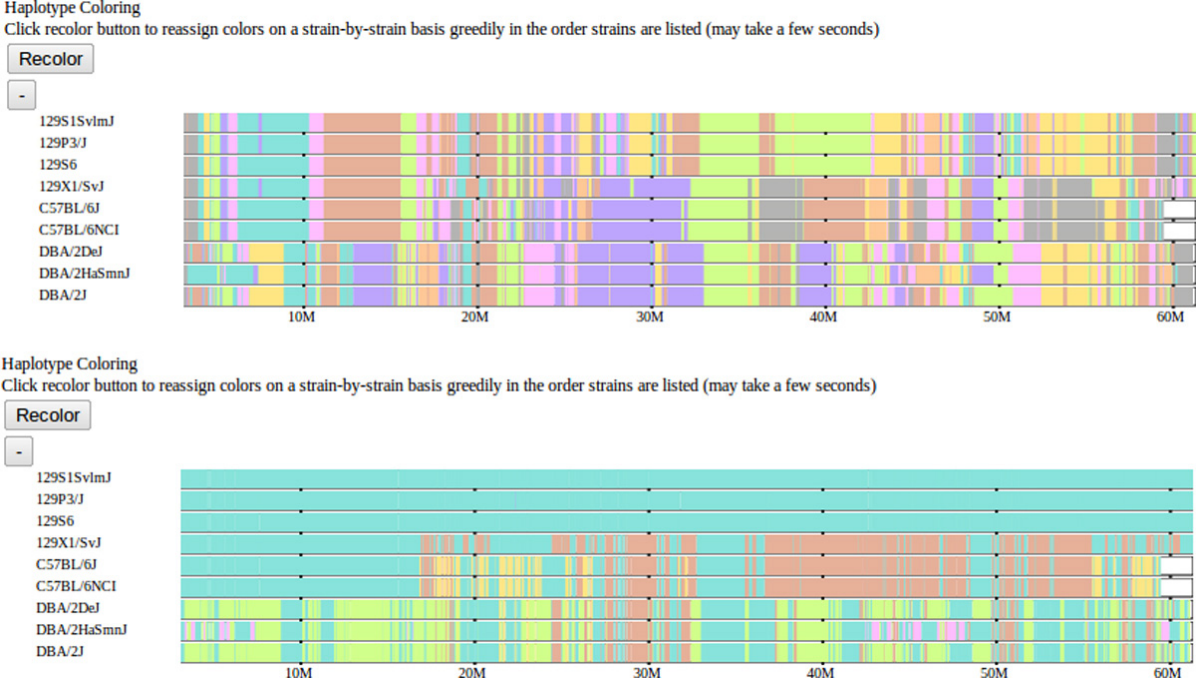
**Haplotype coloring.** The default haplotype block mosaic, which minimizes total color transitions across the entire genome, is shown above. Below is the selected subset of strains recolored according to their displayed order. The topmost strain is assigned a single color and subsequent strains are assigned the same color where their haplotypes match the first strain. A strain is assigned a second color where it does not match the first strain and subsequent strains are assigned the color where they match the new strain but not the first. This process is repeated for all remaining strains in displayed order. This recoloring highlights the haplotype similarities over extended genomic regions (60 Mbases as shown) between the selected strains.

**Figure 5:**
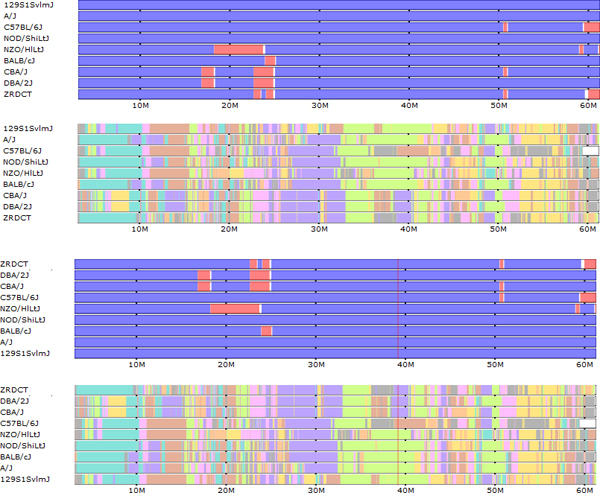
**Sorting.** The top two track groups show subspecific origin and haplotype similarities with strains shown in the default order. The lower two tracks show these tracks after automatic sorting according to the haplotype similarity at the selected cursor position. Strains are sorted by haplotype at the selected position. Strains with the same haplotype are further sorted by adjacent positions until all strains are distinct. The subspecific origin tracks are automatically ordered to match the sorted order as determined by the haplotype color.

Our design focuses on the visualization of multiple simultaneous components from aligned data sets. To this end, we support a multi-row display, where individual data sets are displayed as vertically stacked tracks that be compared vertically, along with derived data tracks which integrate information over the selected subset of samples. We assume collinearity, common local coordinates, of all feature tracks of interest. Our genome browser uses color to allow for more intuitive visualization. Intervals for various data types are displayed as variable-width colored bars across the genome, easily highlighting similarities and differences between genomes by their respective color pattern. To allow users to further customize the visualization to their needs, we support dynamic recoloring of intervals (Figure 4) as well as dynamic sorting of samples at a user-selected position (Figure 5). Dynamic coloring and reordering tools facilitate comparison of features by visually aligning regions where genomes are similar and different.

## Implementation

There are many critical design and resource allocation decisions which arise when handling very large sets of data. In traditional genome browsers, a relatively small amount of data needs to be handled at any one time. Existing browsers only need to handle a single sequence. In order to visualize multiple sequences simultaneously (10 s to 100 s) as in the case of our implementation, it is important to consider different methods for efficient data transfer and visualization. In addition to handling multiple sequences, we also support dynamic visualizations that vary based on the scale and local context. Existing browsers, such as the UCSC Genome Browser [5], do not support large-scale visualization of fine-scale features, like SNPs.

To support faster and more interactive visualization while dealing with remote data, we addressed issues of data transfer and efficiency and how to best allocate the rendering tasks. Our implementation loads data as it is needed into the page using asynchronous requests to the server. To reduce data transfer costs in memory and speed, the page is loaded only once at the beginning of a session and, subsequently, only data is loaded. In addition, visualization and display are handled in the browser by dynamic scripts on the page so that complete images do not have to be transferred from the server. Data rescaling, panning, and drawing are all handled by the client. Requests are made asynchronously so that the tool is available to the user even while new data is transferred.

In order to increase usability as well as allow dynamic content and interaction, we require a set of web-based technologies that would allow wide platform interoperability and dynamic client-server interaction. In addition, to increase ease of development and extensibility, we wanted a framework that was easy to maintain, understand, and use without having to deal with the details of browser compatibility and scripting languages like HTML and Javascript. To meet these objectives, we chose the Pyjamas framework http://pyjs.org, a Python implementation of the Java-based Google Web Toolkit (GWT). This framework allows development using the Python language while outputting browser non-specific HTML and Java- script. To increase efficiency and allow for dynamic client-server interaction, we used AJAX to perform remote procedure calls to retrieve and reformat data as it is needed.

## Results

We have deployed an instance of our visualization tool to aid analysis and interpretation of a recently published Nature Genetics paper [10]. This browser analyzes a set of 100 classical laboratory and 62 wild-derived mouse strains along with 36 wild-caught mice. This study answers open questions regarding the subspecific origin of the laboratory mouse and provides the first detailed view of the haplotype diversity in most common laboratory mouse strains. We use our tool to visualize eight different data types to aid in comparative analysis of these 198 mouse samples.

Several data sets are included to aid in analysis by placing features in a genomic context. We include SNPs from the Mouse Diversity Array [11] used in genotyping the mouse strains. When viewing small sections of the genome, SNPs are displayed individually as vertical bars along the track. In addition, alleles at each SNP for each strain are displayed at fine-scale resolutions overlaying the subspecific origin and haplotype coloring tracks to allow for direct comparison (Figure 3). At coarse-scale resolutions, where SNPs are dense and thus cannot be displayed individually, SNPs are aggregated into a histogram representing the frequency of SNPs within uniformly sized windows. Known genes [12] are displayed in a similar manner. As with the SNPs, genes smaller than a pixel are displayed in a histogram and larger genes are displayed as horizontal bars. In the case where genes overlap, overlapping genes are displayed in additional stacked horizontal tracks.

**Figure 6:**
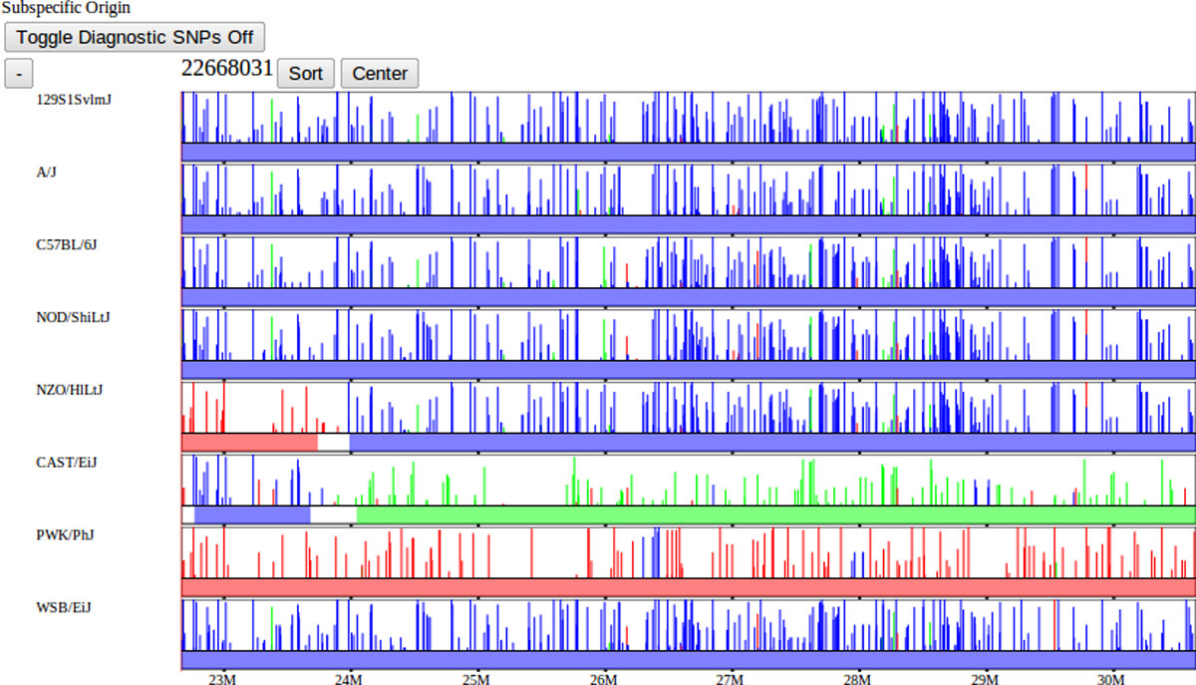
**Diagnostic SNPs.** Subspecific-origin assignments with diagnostic SNPs overlaid within a fine-scale window. Diagnostic SNPs are similarly indicated by color. Diagnostic SNPs were determined by [10] based upon whether each SNP variant was unique to a particular subspecies, and categorized as either fully informative (common to all members of the subspecies) or partially informative (occurring within some members of the subspecies). The SNPs with diagnostic alleles are displayed as individual bars with the height representing the confidence with which that allele indicates the particular subspecies. The subspecific origin intervals are computed using an HMM over the set of diagnostic SNPs for each strain, as described in [10].

A second data set of interest is the local subspecific origin of each sample's genome. The genomes of classical laboratory mouse strains arose through interbreeding of pet mice from three different mouse subspecies. In [10], the mosaic of each genome was determined (Figure 6). Subspecies are assigned to each strain as a mosaic of intervals representing *Mus musculus domesticus*, *Mus musculus musculus*, or *Mus musculus castaneus *subspecies. Subspecies intervals were inferred using a Hidden Markov Model based on a set of diagnostic SNPs. SNPs were assigned their diagnostic status based on each allele's distribution among wild and wild-derived mouse strains of known subspecies. In addition to diagnostic alleles, diagnostic values were also assigned to SNPs based on the distribution VINOs, novel variants not previously identified [13]. Subsequently, genomic regions of mouse strains from unknown subspecies were assigned a subspecies and assigned a confidence based on these diagnostic values. An HMM was used to delineate subspecies intervals across the genome which integrated the diagnostic strength of the SNP markers, a data-error model, and minimized the number of transitions. Subspecific origin is visualized as a horizontal track made up of a mosaic of colored bars representing *domesticus *(blue), *musculus *(red), or *castaneus *(green) regions for each selected strain. At fine scales, diagnostic SNPs are shown above the subspecies assignment for each strain, the height and color of the bar represent the relative diagnostic value and implied subspecies, respectively (Figure 6).

**Figure 7:**
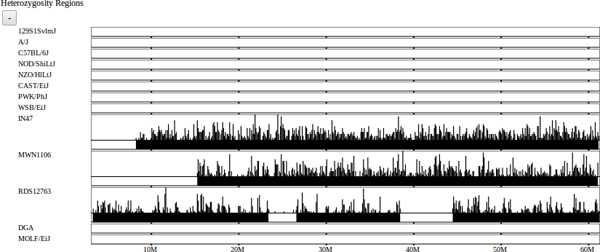
**Heterozygosity.** The heterozygosity track highlights heterozygous regions of the genome, which occur primarily in outbred and wild strains. Similar to SNPs, independent heterozygous calls are represented as a histogram at course scales and individual loci at finer scales. Blocks of heterozygosity are inferred from the individual calls using an HMM, and represent suspected outbred regions. Classical laboratory and wild-derived strains are largely inbred however they may maintain small regions of residual heterozygosity.

Another data set annotates regions of heterozygosity, which are visualized for each selected strain (Figure 7). This is particularly important for outbred and wild-caught mice since laboratory mouse strains have little or no heterozygosity. Heterozygosity is displayed as a mosaic of intervals representing inbred and heterozygous regions in addition to an individual locus-based visualization like the SNP tracks. The locus-based visualization is similar to the SNP visualization, displaying individual heterozygous alleles at fine scales and a histogram representation at coarse scales. The heterozygous block visualization is a computed track using a method similar to the subspecific origin HMM to detect large heterozygous regions.

Genome mosaic representations, such as subspecific origins and heterozygous regions, are useful for revealing the evolutionary history or identifying more recent introgressions between mouse strains. Existing laboratory and wild-derived strains are a mosaic of ancestral genomes that were selected for desired traits and subsequently inbred. Our genome browser provides the first tool for exploring this genomic diversity at both a high level and at a fine scale.

For classical laboratory strains, several additional data tracks are displayed to show local variation and haplotype structure. These data include a mosaic of possibly overlapping intervals or compatible haplotype blocks that show no evidence of recombination. Within these blocks, we dynamically compute identity-by-descent between the selected set of strains. We introduce an innovative visualization of haplotype identity among classical laboratory strains based on these compatible blocks. Lastly, local phylogeny trees can be displayed for each interval.

**Figure 8:**
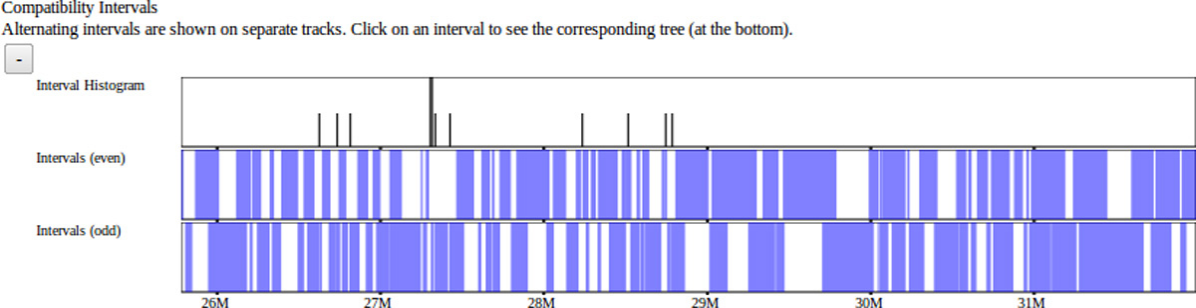
**Compatible intervals.** Intervals larger than the current pixel resolution are displayed as blue bars while the density of smaller intervals are represented as a histogram. Compatible intervals are computed as maximal overlapping 4-gamete compatible regions across the genome. Such intervals have the property that no more than 2 adjacent intervals can overlap. Overlapping "even" and "odd" intervals are displayed on alternating stacked tracks.

We include a visualization of intervals that show no evidence of historical recombination [14] among the classical laboratory strains (Figure 8). These blocks serve as not only a visualization of their own representing ancestral haplotypes but also serve as a framework over which other data sets and visualizations are based. This visualization is useful when analyzing other data sets in order to place those data in the context relative to these intervals. We expect that significant genomic features, specifically differences between strains, should fall within these breakpoints. Compatible intervals are computed using a maximal-k scan (Wang et al, 2010) over 100 classical laboratory strains, that is, a minimal full covering of maximally sized intervals based on the 4-gamete test. Such intervals each define a unique perfect phylogeny tree based on the set of SDPs within the interval. Intervals can overlap at most with one other interval on each side, so intervals are displayed as horizontal bars in one of two stacked horizontal tracks. At a scale too large to display individual intervals, the density of intervals is once again displayed as a histogram.

**Figure 9:**
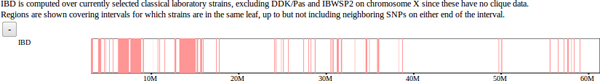
**Identity-by-descent.** The dynamic IBD track shows intervals of sequence identity computed over the selected subset of strains. Red horizontal bars represent regions of the genome over which all selected strains are substantially identical. IBD is computed from the haplotype group assignments for each interval where strains in the same haplotype group are considered IBD. Regions of IBD are displayed where all selected strains are in the same haplotype group over consecutive compatible intervals. This visualization is computed on the fly according to the user-selected subset of strains.

Intervals of sequence identity are computed dynamically based on where the subset of selected strains share a common haplotype (Figure 9). Strains are divided into haplotype identity groups within each compatible interval based on sequence similarity (see [10]). We compute intervals of identity by descent (IBD) over the user-selected set of strains on the fly. We consider a region IBD if all selected strains are in the same haplotype group over that region. Consecutive intervals of IBD are combined into larger blocks in part to reduce the data transfer size. As we will see in the following data description, regions of IBD should correspond directly to identical haplotype coloring patterns.

We also support a method for exploring the extent of shared haplotypes among the selected strains. Blocks of color are used to depict haplotype similarity. Colors are assigned and reused so that transitions are minimized. This provides a relative comparison of strain similarities (Figure 4). At any position along the genome, the haplotype identity among the displayed strains can be understood visually as dividing strains into haplotype groups according to their color such that strains with substantially identical haplotypes are the same color. Over larger regions, haplotype identity is represented by a shared color pattern.

Initial haplotype colors are precomputed for all classical laboratory strains, leading to frequent color/haplotype changes at a genomic scale. When viewing only a small sample of strains, this coloring can be simplified, essentially changing colors only when there are haplotype group changes among the selected strains. An interactive aspect of this visualization is that the colors can be dynamically reassigned according to the order of the selected strains such that colors are assigned in descending order (Figure 4). The topmost displayed strains is assigned a single color across the genome. The second strain is assigned the color of the previous strain where its haplotype matches the first and a second color where it differs. This process is repeated for subsequent strains. This has the effect of, for example, highlighting all regions where the first selected sample shares a haplotype with subsequent samples by using the same color. In this way, the haplotype coloring scheme can be substantially simplified for a small sample of strains allowing more intuitive analysis. A generic feature of the browser is that strain tracks can be dragged vertically to reorder their position within a track group, allowing the coloring order to be customized for the analysis required.

A final interactive tool facilitates similarity analysis at a particular position by allowing sorting of tracks within all groups at a user-selected position within the displayed genomic window. Strains are sorted vertically according to the haplotype coloring at the selected position such that strains with identical haplotypes are grouped together. In addition, strains are further sorted according to their haplotypes at increasingly distant positions radiating in both directions from the selected position until either the edge of the displayed window is reached or all strains are distinct.

**Figure 10:**
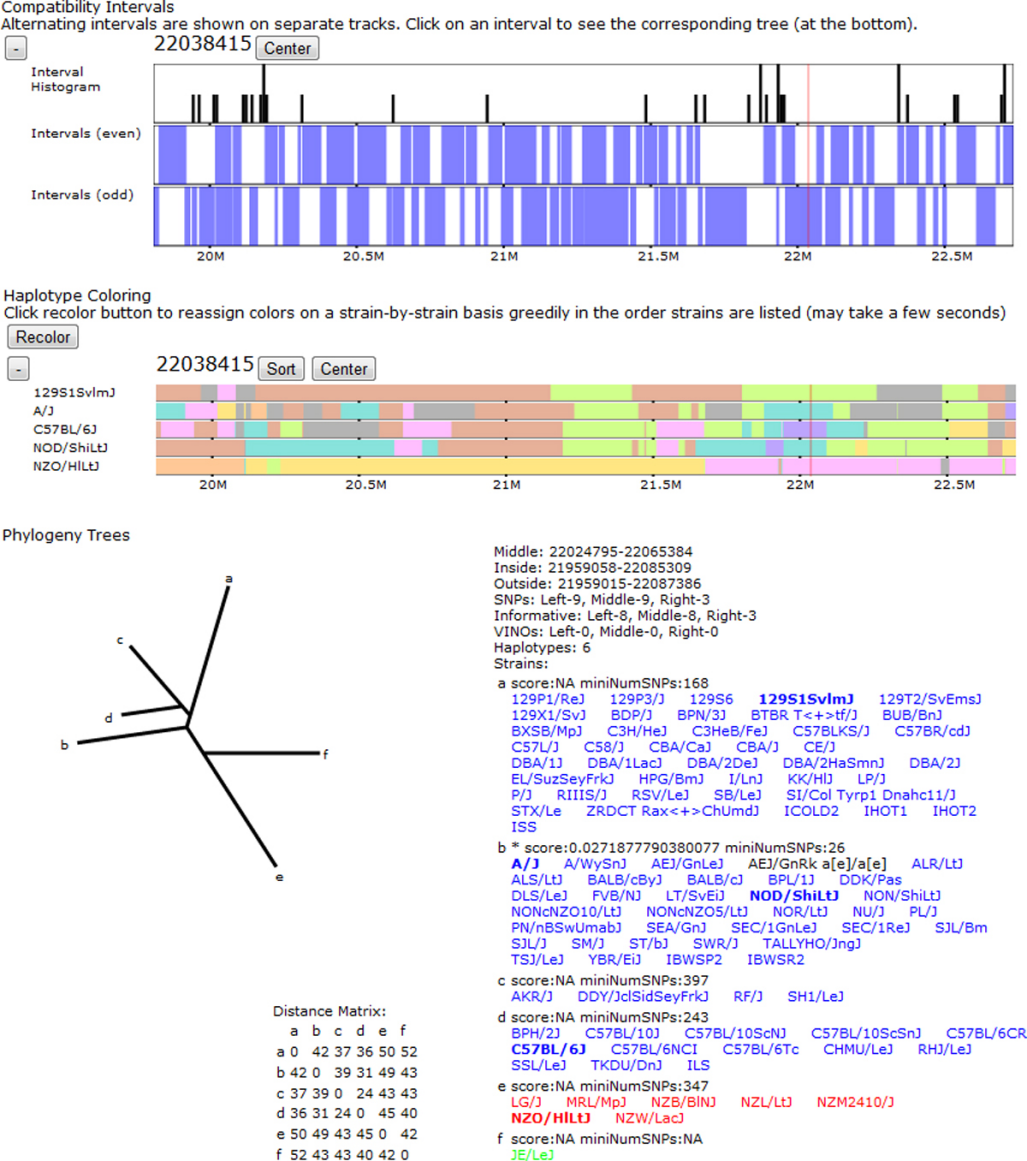
**Local phylogenetic tree.** The compatible intervals and haplotype coloring are shown for a small region. A local phylogeny tree is also shown for the highlighted interval (denoted by the vertical red bar along the compatible interval track). The local phylogeny tree visualization includes the tree structure, size and location annotation for the interval the tree covers, the leaf descriptions including the strains in each leaf, and the distance matrix used to perform the neighbor-joining between leaves. Letters at the leaves of the phylogeny tree denote nodes that contain strains. The leaf descriptions show the corresponding node letter, a confidence score, and number of supporting SNPs along with the set of strains in that leaf. Each strain is colored according to its assigned subspecific origin within the tree's interval and the strains in the currently selected subset are shown as bold to allow the user to quickly identify them.

Lastly, local phylogenetic trees can be displayed by selecting a compatible interval of interest within the genome (Figure 10). A tree is computed within the interval based on neighbor-joining on haplotype similarity. Selected strains are highlighted according to the haplotype group they fall in, corresponding to a leaf in the tree structure. In contrast to the haplotype identity and IBD tracks, the phylogenetic trees show relative differences and possible ancestral relationships between similar haplotype groups rather than simply the group membership. Strain names are colored according to their subspecific origin to show the relationship between subspecies assignment and tree structure.

## Conclusions

An instance of our genome browser and its dynamic analysis methods has been deployed to display results of our recent publication [10] at http://msub.csbio.unc.edu. It is continually used in comparative genome analyses of the mouse genomes presented. In the past twelve months of our tool's availability, we have had over 4000 users make almost 50,000 queries. The tool is used by researchers to perform comparative analysis between 198 common mouse strains. Our tool is particularly well suited for selecting and partitioning strains while simultaneously considering phenotype variation as it relates to a given gene or genomic region. A recent focus of our browser has been to explore the predictive power of our local phylogeny and haplotype assignments. Local comparative genomic analysis has been shown to be particularly effective in predicting disease susceptibility and other phenotypic states of the available set of mouse strains given the known state of a small sample. Our notion of sequence similarity has also been used to inform genotype imputation by constructing a haplotype mosaic [15]. Work is continuing to enhances the browser's support in this area.

**Figure 11:**
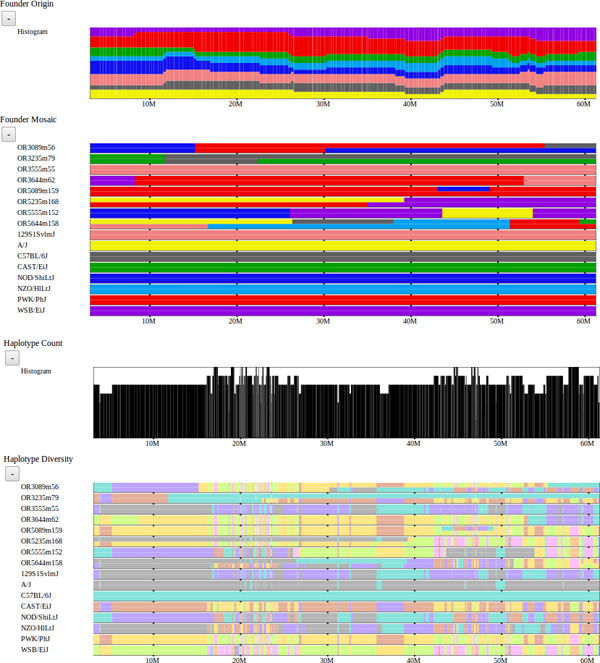
**Collaborative Cross Viewer.** A sample of the data tracks available in the Collaborative Cross Viewer http://csbio.unc.edu/CCstatus/?run=CCV. The viewer supports analysis and visualization of 458 emerging CC lines and the 8 founder strains [10]. From top to bottom, the CC founder origin histogram and mosaic for a subset of lines is shown, then a histogram of the number of unique haplotypes and the haplotype diversity for the selected strains. The founder origins indicate the likely founder haplotypes inherited across the genome for the CC lines since the CC lines are a mosaic of the 8 founders. Note that many of these lines are not yet fully inbred, so there exist regions of heterozygosity as well as homozygosity. The haplotype diversity is similar to the notion of haplotype intervals described for the Mouse Phylogeny Viewer.

We have also implemented another version of our framework to support preliminary analysis of the emerging Collaborative Cross (CC) lines [16]. This browser supports analysis of 458 mouse lines in the Collaborative Cross in various stages of inbreeding as well as the 8 inbred founder strains [17]. Visualized data sets include the assigned founder mosaic for each CC line, subspecific origin, haplotype diversity, and local phylogenetic trees. This resource is available at http://csbio.unc.edu/CCstatus/?run=CCV and is being used extensively by the Collaborative Cross Consortium and others to analyze these data. Figure 11 shows a snapshot of this tool.

There are many technical as well as structural improvements that can be made in the future to make our browser more useful, general, and effective for visualization and analysis of multiple genome data. Although the browser is constructed in a modular format, separating our data from the browser itself, to add new user-specified data types or novel visualizations requires changes to the source code and recompiling. In order to support a larger range of users and wider adoption, it is possible to add a simple web-based user interface for adding new tracks and visualizations within the existing framework. We present our genome browser's application to a specific data set here, but it is suitable to other organisms and other data types where comparative analysis of multiple genomes is useful. Likewise, we could provide an API for custom analysis of the existing data set. A more fundamental improvement we would like to make is to support local structural variations, such as insertions, deletions, repeats, and translocations, where the compared genomes are not strictly collinear as we assume. Even samples of the same subspecies can have small scale copy-number variations and are not strictly collinear. Assessing these differences is an important part of local haplotype and phylogeny analysis.

## Competing interests

The authors declare that they have no competing interests.

## Authors' contributions

JW developed the methods, designed and implemented the tool, and drafted the manuscript. FPMV conceived of the resource and provided the data. LM participated in design of the methods and tool and helped draft the manuscript. All authors read and approved the final manuscript.
